# Editorial: Oral Microbiome and Inflammation Connection to Systemic Health

**DOI:** 10.3389/fcimb.2021.780182

**Published:** 2021-11-02

**Authors:** Loreto Abusleme, Ana Carolina Morandini, Tomomi Hashizume-Takizawa, Sinem Esra Sahingur

**Affiliations:** ^1^ Laboratory of Oral Microbiology, Department of Pathology and Oral Medicine, Faculty of Dentistry, University of Chile, Santiago, Chile; ^2^ Laboratory for Craniofacial Translational Research, Faculty of Dentistry, University of Chile, Santiago, Chile; ^3^ Department of Oral Biology and Diagnostic Sciences, Dental College of Georgia at Augusta University, GA, United States; ^4^ Department of Periodontics, Dental College of Georgia at Augusta University, GA, United States; ^5^ Department of Microbiology and Immunology, Nihon University School of Dentistry at Matsudo, Chiba, Japan; ^6^ Department of Periodontics, School of Dental Medicine, University of Pennsylvania, PA, United States

**Keywords:** oral bacteria, systemic diseases, inflammation, oral microbiome, dysbiosis

In recent years, our current understanding of the interplay between the oral microbiome and the host immune response has expanded considerably, becoming increasingly complex. Significant progress has been made towards studying how host microbial interactions can shape the clinical outcomes in health and disease. We now appreciate that these microbes are also taking part of diverse microbial assemblies inhabiting the oral cavity. One of the critical factors that has contributed to this knowledge is the wide utilization of next-generation sequencing techniques, which have allowed to unravel the complexity of oral microbial communities from a more ecological perspective. For instance, the study by Saito et al. utilizing a very large data-set of over 1,000 oral samples, have uncovered relevant differences between salivary and tooth-associated plaque microbial communities. They also showed that microbial diversity and abundance of *Treponema* and *Selenomonas* were increased as periodontal disease progresses and describe novel bacterial interactions during disease.

The influence of environmental factors on the oral microbiome has been a critical question in the field that continues to be interrogated. The study by Jia et al. examined the effect of cigarette smoking on the oral microbiota in a Chinese cohort of 316 healthy subjects. The results showed that smokers had significantly higher diversity and select bacterial species were associated with cigarette smoking. Further analyses showed increased levels of *Actinomyces* and *Veillonella* and expression of acid production-related pathways in smokers. The study concluded that cigarette smoking may affect oral health by creating an acidic environment that alters bacterial abundance and metabolic function.

We have also gained a much broader view of the importance of the oral microbiome and how its dysbiosis may play a crucial role in the pathogenesis of not only local diseases but in several systemic conditions. Huang et al. presented relevant data of the salivary microbiome of 293 patients with progressive histological stages of gastric carcinogenesis. Patients with gastric cancer had increased pro-inflammatory bacteria; whereas the genera *Haemophilus* and *Neisseria* were decreased, which may lead to accumulation of carcinogens. Their findings suggest that oral bacteria may be involved in the pathogenesis of gastric carcinogenesis and propose potential diagnostic bacterial biomarkers for gastric cancer. Examining other systemic condition, He et al. showed how the oral microbial dysbiosis was associated with the pathogenesis of Immunoglobin A Nephropathy (IgAN), the most common type of glomerulonephritis. Data include the profile of the most abundant oral microbiota species in diseased patients *versus* controls indicating the shared and specific species between the two groups. The authors also showed correlative studies between oral microbiota and clinical sub-phenotypes of the disease defining predictive functional profiles that may be more associated with IgAN.

Translocation of oral microorganisms to distant sites is also another critical aspect that may constitute a direct mechanism by which select oral bacteria impact systemic health. In the study by Bordagaray et al. the authors reported the potential dissemination of bacteria associated with apical periodontitis to the bloodstream. *Porphyromonas* spp. were frequently detected in tissues and root canals exudates from apical lesions. Total bacteria and *P. endodontalis*, but not *P. gingivalis*, were detected in peripheral blood mononuclear cells (PBMCs) from patients, suggesting that *P. endodontalis* may disseminate from the apical lesion to the systemic bloodstream by PBMCs. On a related topic, the relationship of oral bacteria and the overall bone metabolism is starting to be explored. Hirohashi et al. addressed a technical issue in their study, regarding the possibility to use tetracycline as a fluorescent dye to evaluate bone loss caused by the tetracycline-sensitive oral bacteria, *Streptococcus mutans*. Their findings revealed that tetracycline can be utilized in the measurement of bone loss induced by drug-sensitive bacteria, and that *S. mutans* triggers a reduction of bone mass possibly by reducing bone formation activity.

The study of host-microbial interactions between oral microorganisms and stromal or immune cells is at the center of the mechanistic understanding of how select oral pathobionts play a role in the pathogenesis of oral and systemic conditions. Oral bacteria associated with periodontal diseases have been the most thoroughly characterized in a variety of pathological settings. *Porphyromonas gingivalis* is a prominent example, and the study by Elsayed et al. assessed the role of this organism in the vulnerability to senescence in human primary isolated gingival keratinocytes and bone marrow-derived dendritic cells (DCs) from young (yDCs) and old (oDCs) mice. The authors found increased senescence, impaired maturation of DCs and proliferation of CD4^+^ T cells upon oDCs or yDCs+Pg stimulation. A characterization of exosomes of DCs (control and DCs+Pg) revealed a differential regulation of miRNAs in the *Pg* challenged group alongside an enrichment of the fimbrial adhesin protein mfa1 and pro-inflammatory cytokines. The study concluded that *P. gingivalis* can induce premature senescence through direct cellular invasion and by stimulating secretion of exosomes that promote paracrine immune senescence in bystander cells. Focusing on another periodontal organism, *Treponema denticola*, the study by Malone et al. evaluated the influence of this bacterium on the actin filaments of periodontal ligament (PDL) cells. The authors elegantly described mechanistic interactions between *T. denticola* and human PDL cells, showing that this periodontopathogen drives a pathological response characterized by upregulation of RASA4, depolymerization of actin filaments, and a subsequent increase in MMP-2 activity. Authors speculate that these contributions shed light on new potential targets for *T. denticola*-PDL interactions that may influence periodontal tissue homeostasis. The study of Li et al. examined the interplay of another periodontal bacteria, *Fusobacterium nucleatum* in combination with a recognized respiratory pathogen, *Pseudomonas aeruginosa*, and analyzed how these two organisms interacted with pulmonary epithelial cells. Co-infection of both organisms or pre-treatment with *F. nucleatum* resulted in increased bacterial invasion and inflammatory cytotoxicity of pulmonary epithelial cells. These findings provide novel insights on the mechanisms behind the deterioration of lung function associated to these two organisms.

We would like to thank all authors in this collection for their valuable contributions. We hope this Research Topic continues to stimulate the generation of new knowledge dedicated to unravel influence of the oral microbiome on systemic health.

## Author Contributions

All authors listed have made a substantial, direct, and intellectual contribution to the work and approved it for publication.

## Funding

This work was funded in part by ANID/Chilean National Fund for Scientific and Technologic Development (FONDECYT), grant #11180505 (to LA), and grants R01DE025037 and R01DE027374 from the National Institute of Dental and Craniofacial Research/National Institutes of Health (to SES).

## Conflict of Interest

The authors declare that the research was conducted in the absence of any commercial or financial relationships that could be construed as a potential conflict of interest.

## Publisher’s Note

All claims expressed in this article are solely those of the authors and do not necessarily represent those of their affiliated organizations, or those of the publisher, the editors and the reviewers. Any product that may be evaluated in this article, or claim that may be made by its manufacturer, is not guaranteed or endorsed by the publisher.

